# Corticosteroid use and bone health management for Duchenne muscular dystrophy in South Korea

**DOI:** 10.1038/s41598-022-15510-1

**Published:** 2022-07-04

**Authors:** Jin A Yoon, Ho Eun Park, Jinmi Kim, Jungmin Son, Yong Beom Shin

**Affiliations:** 1 Department of Rehabilitation Medicine, Pusan National University School of Medicine and Biomedical Research Institute, Pusan National University Hospital, 179 Gudeok-Ro Seo-Gu, Busan, 602-739 Republic of Korea; 2 Department of Biostatistics, Clinical Trial Center, Biomedical Research Institute, Pusan National University Hospital, Busan, Korea

**Keywords:** Neuromuscular disease, Rehabilitation

## Abstract

This study aimed to determine the current corticosteroid use and bone health management status of patients with Duchenne muscular dystrophy (DMD) in South Korea. This is a national population-based study utilized information from the databased of Korean National Health Insurance Database. Database regarding bone status, spine radiography findings, bone mineral density, and laboratory test results were obtained, as well as the proportion of patients with spine and lower limb prostheses, occurrence of scoliosis, and age at scoliosis surgery. Deflazacort dose in the ambulant group (aged < 15 years) increased by age and year. The maintenance dose of prednisolone and deflazacort for the 15–19 years group decreased by year. Among the patients, 12.47% underwent spine radiography, 23.11% underwent dual-energy X-ray absorptiometry, and 22.7% underwent vitamin D tests. Moreover, 40.9% of the patients were prescribed vitamin D at a mean age of 14.6 ± 6.1 years, while 10.22% were prescribed bisphosphonate at 17.92 ± 3.4 years. Further, 16.1% of the patients underwent posterior spinal instrumentation and fusion at 14.4 ± 2.3 years and 5.3% underwent anterior spinal instrumentation and fusion at 14.4 ± 2.3 years. Ankle–foot orthosis and spine orthosis prescriptions were noted in 4.91% and 1.84% of patients, respectively. In this our study, the current corticosteroid use and bone health management status of DMD in South Korea has been presented. The dose prescription for corticosteroid and bone health monitoring did not reach to current recommendation.

## Introduction

Duchenne muscular dystrophy (DMD) is the most common childhood muscular dystrophy with a worldwide incidence of 1 in 5000 live male births^1^. A 2007 epidemiological investigation conducted by the Centers for Disease Control in South Korea reported an annual number of 3459 patients with muscular dystrophy, with the highest proportion shown to have Duchenne or Becker muscular dystrophy, a subtype with severe clinical symptoms, whereas the proportion of those with muscular dystrophy was presumed to be 30–40%^2^. However, no study has yet provided an established database or clinical information regarding patients with DMD in South Korea.

In the past few decades, most patients with DMD died in their 20 s owing to respiratory failure or cardiomyopathy^3,4^. The use of glucocorticoids has been shown to have multiple benefits, including prolonged life expectancy and delayed loss of ambulation, respiratory dependence, and the onset of scoliosis^5,6^. The potential beneficial effects of corticosteroid include inhibition of muscle proteolysis, stimulation of myoblast proliferation, stabilization of muscle fibre membranes and so on^7^. According to the care consideration mentioned in the guidelines for glucocorticoid use DMD care recommendations updated in 2010^8,9^ and 2018^10^, the drug should be used before a substantial physical decline occurs. However, caution is required for adverse effect of long-term clinical use of use of corticosteroid. These include loss of bone mineral density, increased bone fracture, cataracts, weight gain, growth failure^7^ Therefore, appropriate prescription of corticosteroid and monitoring the relevant side effects including bone health problem is important to achieve the long-term functional benefit of corticosteroid.

Otherwise, a recent expert survey in Asian countries showed that approximately 20% of clinicians did not use steroids owing to side effects^11^, and the regimen also differed in each country. These uncertainties increase the risk of undertreatment or overtreatment, which could confound the results of clinical trials of innovative therapies. There is yet to be a study providing an established database or clinical information regarding patients with DMD in South Korea. Further, no study has examined the status of guideline-recommended corticosteroid use and bone health management in South Korea.

Therefore, the present study aimed to determine the current status of corticosteroid use and bone health management of patients with DMD in South Korea based on the Korean National Health Insurance Database as well as to provide fundamental data for creating and standardizing the national guidelines for DMD treatment among clinicians in South Korea.

## Materials and methods

This is a national population-based study utilized information from the database of Korean National Health Insurance Database. The present study aimed to determine information such as the current corticosteroid use and the related bone health management status of patients with DMD in South Korea and to provide fundamental data for creating and standardizing the national guidelines for DMD treatment in the future.

### Identification of subjects

**Figure 1:**
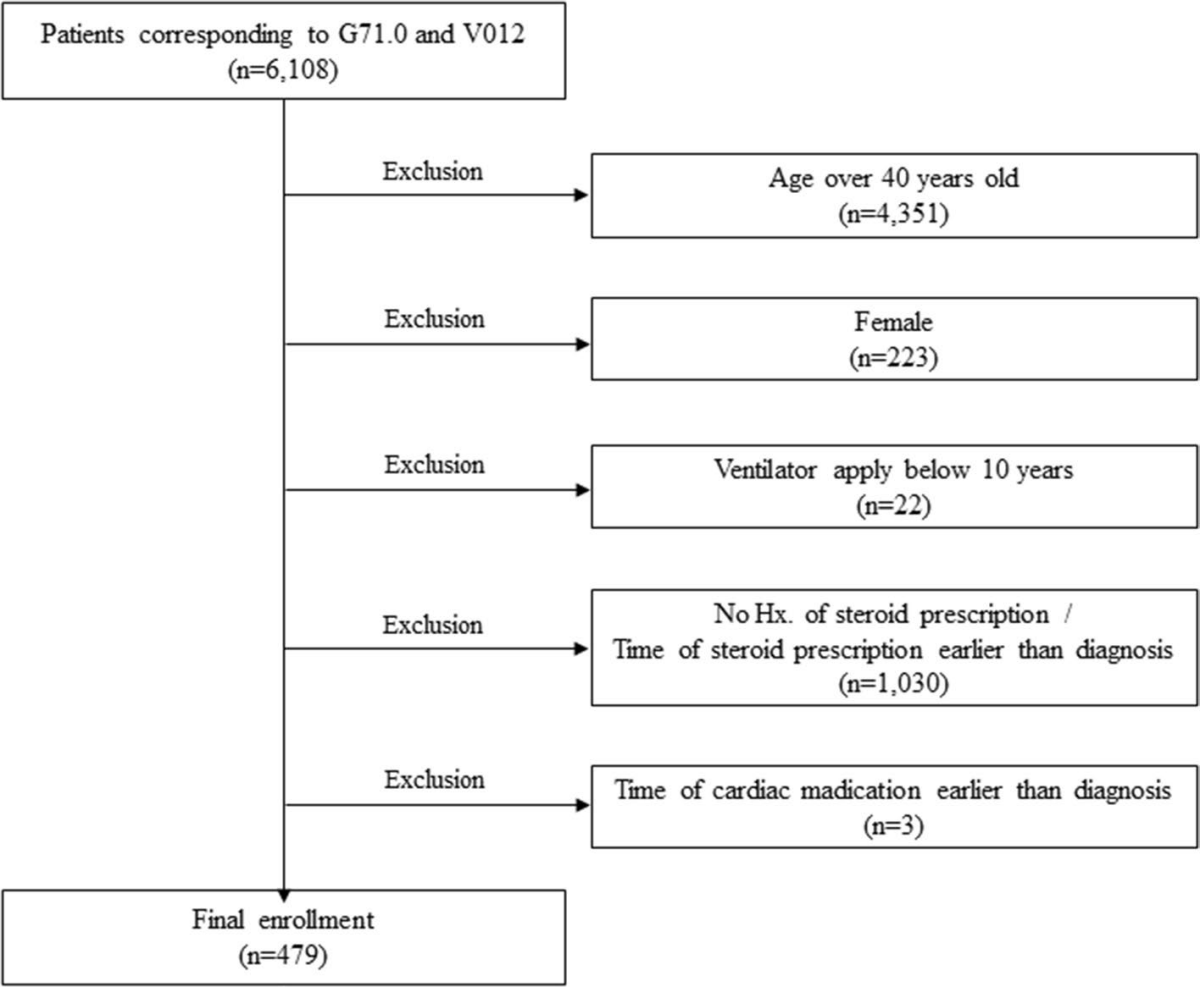
Flow diagram of patient inclusion and exclusion.

From this database, the data of patients corresponding to G71.0, Muscular dystrophy (MD) and the V012, special code that extends health insurance coverage to patients with rare intractable diseases according to the *Tenth Revision of the International Classification of Diseases* main diagnosis and sub-diagnosis were extracted. To screen the patients with DMD, the operational definition was set as a male patient under 40 years old who had been diagnosed before the age of 10 years. To exclude other types of MD, patients who had been prescribed steroids were selectively extracted according to the recent recommendation on the current status of steroid prescription for DMD patients in South Korea^12^ to construct a database. In addition, patients who did not demonstrate the typical clinical course: female, age over 40 years old, ventilator apply below 10 years, time of corticosteroid and cardiac medications earlier than diagnosis were excluded from the study. The wash-out period was 7 years. The patient screening algorithm using the National Health Insurance claims data is presented in Fig. 1.

### Data acquisition and analysis

National Health Insurance Service (NHIS), which is the only health insurance system in South Korea has been granted to access to health utilization information by researchers. All people in South Korea are eligible for coverage under the NHIS. The Health Insurance Review and Assessment (HIRA) database of healthcare service information including diagnosis, procedures and prescriptions was provided to the researchers by the appropriate guidelines of NHIS. We analyzed the number of patients at intervals of 5 years, and if the dates of diagnosis and death followed the onset of recuperation, the mortality date was verified. Regarding the use of corticosteroids, whether the patient had been given a steroid prescription, the percentage of prednisolone or deflazacort among all prescriptions, the patient’s age when the prescription was given, and the dose prescribed for each age group were examined.

Regarding bone status, spine radiography, dual-energy X-ray absorptiometry (DXA), and vitamin D laboratory tests were performed, and the interval of these tests was determined. The age when the spine and lower limb prostheses were prescribed was analyzed. In addition, to estimate when the loss of gait ability occurred, we checked when a wheelchair prescription was issued. The data of orthosis prescription were extracted from the benefits payment table of 2016–2018 when orthosis prescription was included for insurance benefit. In addition, whether scoliosis surgery had been performed and the age at which the surgery was performed were checked.

To validate extracted data, the number of patients was extracted from the database using codes for the 5 specified local tertiary hospitals and nursing facilities established under special purposes that were thought to be research institutions in 2018. This number was compared with the number of patients screened using the diagnostic code for DMD and those who received corticosteroid treatment from this cohort.

### Ethics statement

The study was approved by the Institutional Review Board of the Pusan National University Hospital (approval number 1907-008-080) and the National Health Insurance Service of Korea (approval number REQ0000030402). The requirement for informed consent was waived because secondary data were used. All methods were performed in accordance with the relevant guidelines and regulations.

## Results

From 2002 to 2018, approximately 479 patients met the diagnostic criteria (Fig. 1). Among the DMD patients with corticosteroid prescription, the late non-ambulatory patients (age > 20 years) increased over the years. A total of 52 (10.8%) patients died, and the mean age at death increased by year (Table 1). For validation, the data of 126 patients in 2018 from the 5 specified local tertiary hospital facilities were extracted. The same number of DMD patients who received corticosteroid treatment at 5 hospitals in 2018 were identified using medical record review.

**Table 1:** Number of DMD patients with corticosteroid prescription and age of death by year.

(A) Number of DMD patients with corticosteroid prescription																	
Age	2002	2003	2004	2005	2006	2007	2008	2009	2010	2011	2012	2013	2014	2015	2016	2017	2018
1–4	25	32	35	35	42	50	47	45	48	42	40	34	40	36	35	30	19
5–9	72	94	113	135	126	113	114	107	91	93	92	90	78	77	72	72	62
10–14	11	19	42	71	113	137	161	163	167	143	124	118	114	96	96	94	92
15–19	0	0	0	0	0	11	18	41	69	110	131	154	154	158	134	113	105
20–24	0	0	0	0	0	0	0	0	0	0	10	17	38	65	102	124	141
> 25	0	0	0	0	0	0	0	0	0	0	0	0	0	0	0	10	18
Total	109	145	190	242	283	314	342	357	375	388	398	415	426	432	440	443	433

(B) Mean age of death by year																	
year	2009	2010	2011	2012	2013	2014	2015	2016	2017	2018							
Age	12.3 ± 5.0	14.2 ± 1.7	14.2 ± 1.7	14.0 ± 2.8	14.4 ± 7.4	13.0 ± 4.0	17.6 ± 4.5	18.0 ± 2.8	19.1 ± 3.2	20.4 ± 4.2							

**Figure 2:**
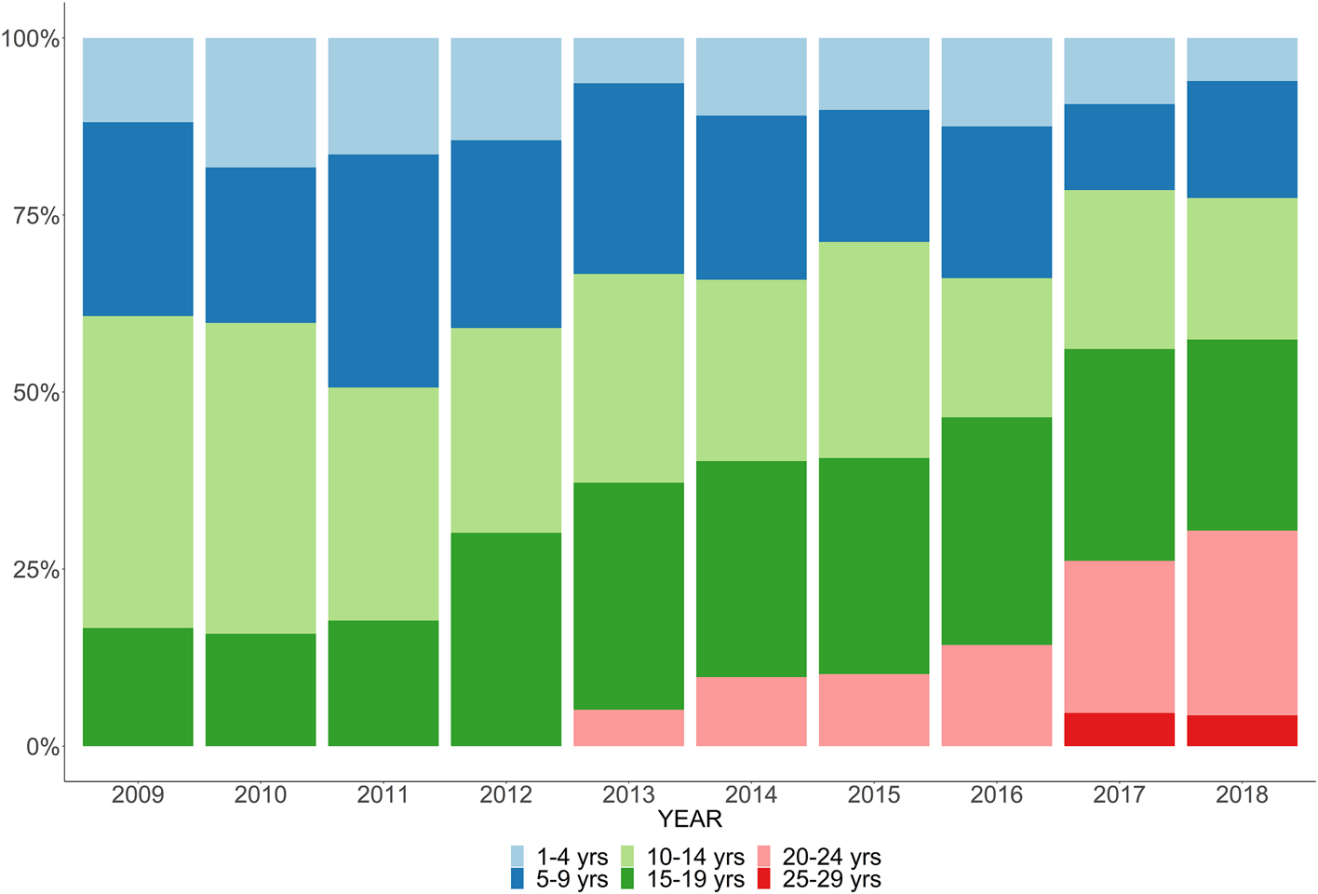
Corticosteroid use in South Korea based on the National Health Information Database.

**Figure 3:**
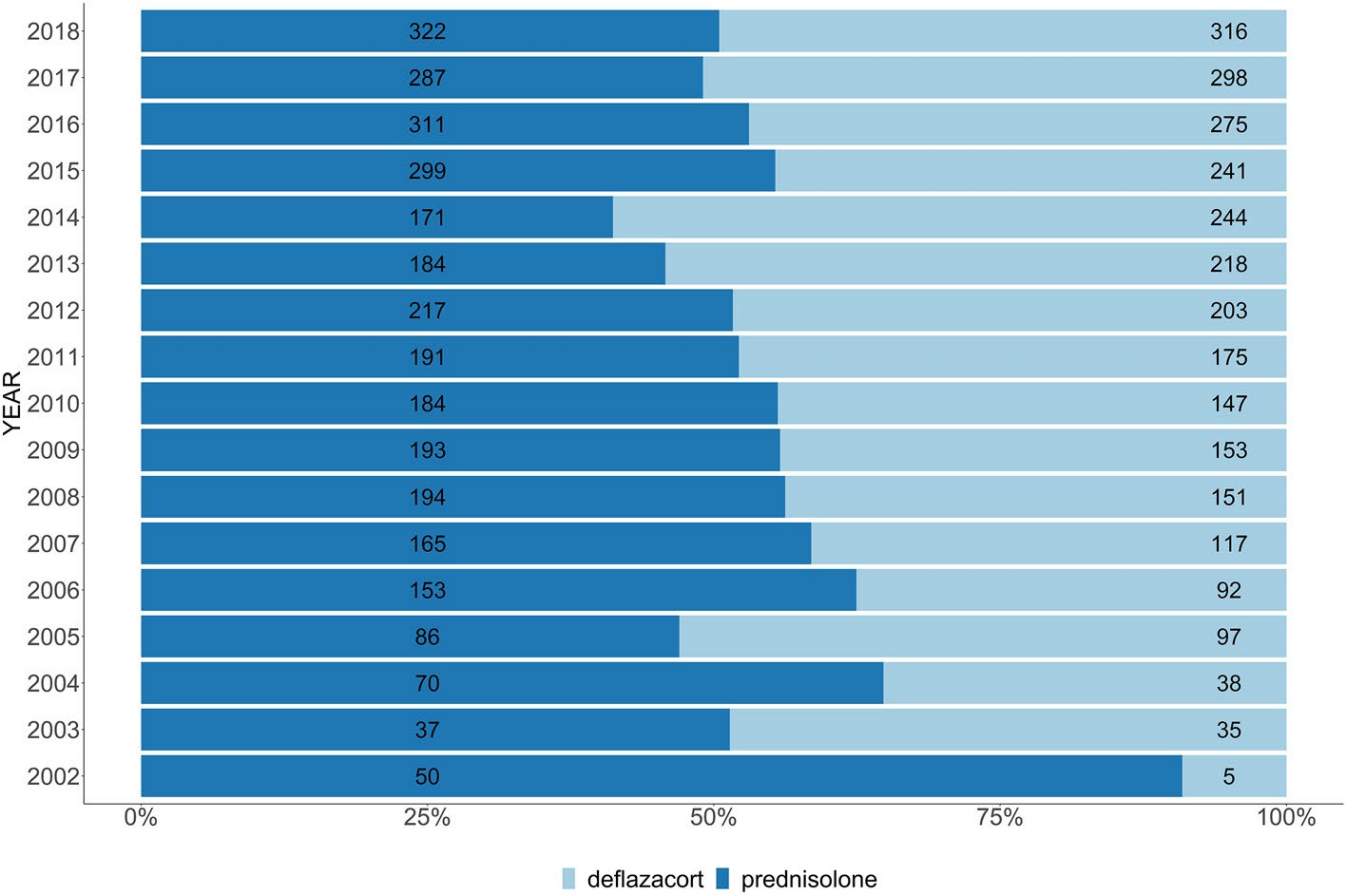
Proportion of deflazacort and prednisolone prescription in 2002–2018.

**Figure 4:**
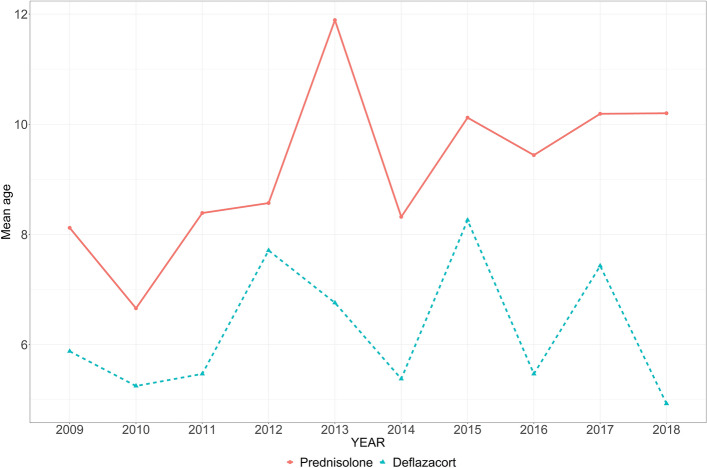
Mean age of patients prescribed corticosteroids in 2009–2018.

**Figure 5:**
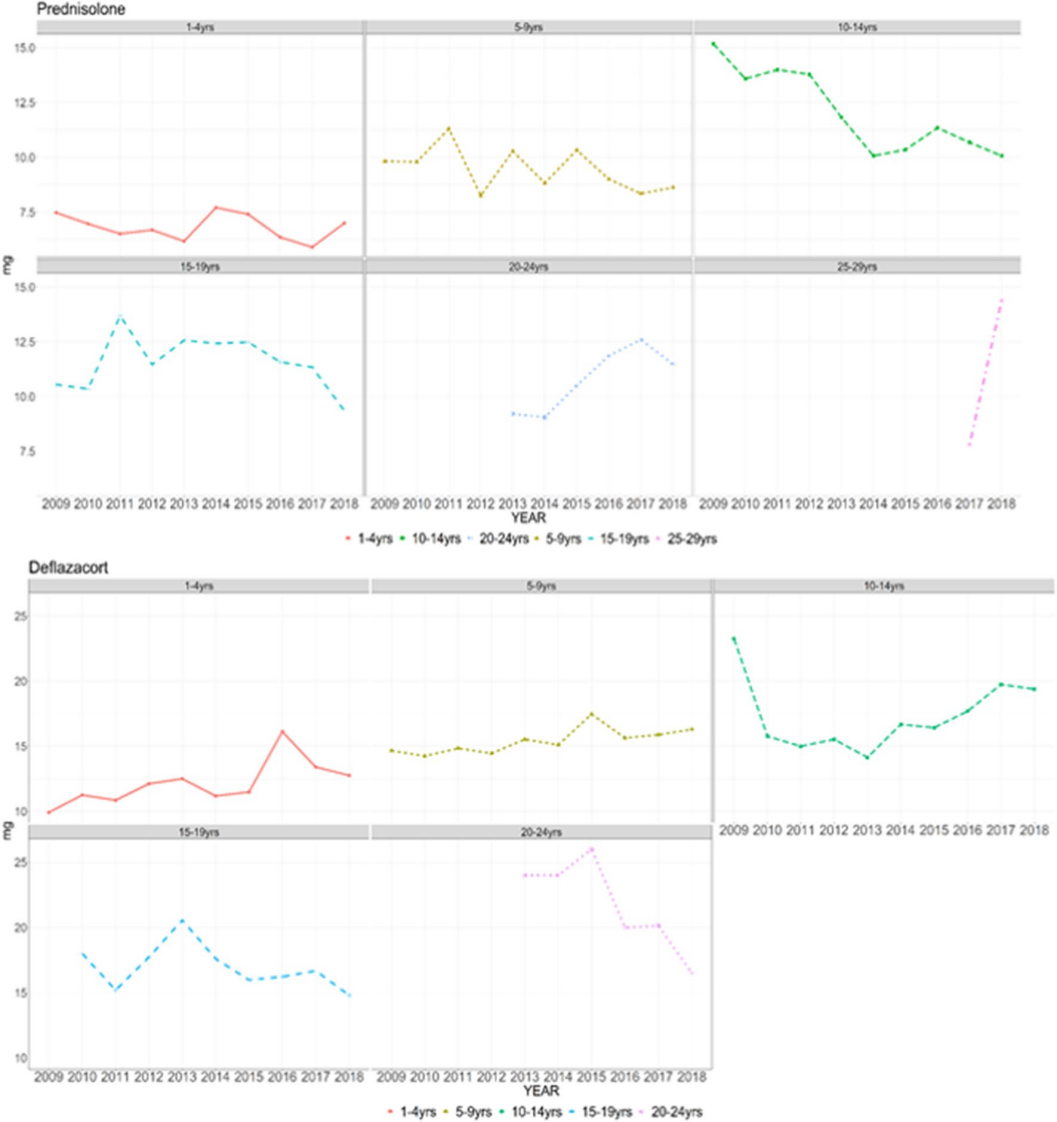
Mean dose of corticosteroid prescription per day in 2009–2018.

The mean age of wheelchair prescription to predict the time of loss of ambulation was 11.9 ± 3.4 years. The corticosteroid prescription by age and year from 2009 to 2018 showed proportion of maintenance corticosteroid therapy for non-ambulant patients (age > 20 years) increased over the years (Fig. 2). The proportions of patients with deflazacort and prednisolone prescriptions are presented in Fig. 3 and showed a similar ratio recently. The mean age of initial corticosteroid prescription by year showed younger in deflazacort compared to prednisolone (Fig. 4). The mean prescription dose per day for prednisolone and deflazacort is shown in Fig. 5. Deflazacort prescription dose for the ambulant group (age > 15 years) increased by age and year. The maintenance dose of prednisolone and deflazacort for those aged 15–19 years decreased by year (Fig. 5).

Among all patients, 65 (13.6%) underwent spine radiography with a 509.2 ± 470.5-day interval, 118 (24.6%) underwent DXA within a 1019.4 ± 903.4-day interval, and 111 (23.2%) underwent vitamin D level tests. Of these patients, 193 (40.3%) had vitamin D prescription at a mean age of 12.5 ± 4.8 years and 48 (10.2%) had bisphosphonate prescription at a mean age of 17.9 ± 3.4 years with a 170.5 ± 245.4-day interval (Table 2A).

**Table 2:** Status of bone health management in the study population ((N = 479).

Characteristics	Total (N = 479)
**(A) Status of bone health evaluation and prescription**	
Spine radiography, *n* (%)	65 (13.6)
Age at spine radiography, years, mean ± SD	13.5 ± 4.8
Interval of spine radiography, day, mean ± SD	509.2 ± 470.5
Dual-energy X-ray absorptiometry (DXA) scan, *n* (%)	118 (24.6)
Age at DXA, years, mean ± SD	13.58 ± 5.1
Interval of DXA, days, mean ± SD	1019.4 ± 903.4
Vitamin D level, *n* (%)	111 (23.2)
Age at vitamin D level measurement, year, mean ± SD	14.6 ± 6.1
Bisphosphonate prescription, *n* (%)	48 (10.2)
Age at bisphosphonate prescription, year, mean ± SD	17.9 ± 3.4
Interval of bisphosphonate prescription, day, mean ± SD	170.5 ± 245.4
Vitamin D prescription, *n* (%)	193 (40.3)
Age at vitamin D prescription, year, mean ± SD	± 4.8
**(B) Orthopedic and surgical management in the study population**	
Ankle–foot orthosis prescription, *n* (%)	22 (4.6)
Spine orthosis prescription, *n* (%)	9 (1.9)
Posterior spinal instrumentation and fusion, *n* (%)	77 (16.1)
Anterior spinal instrumentation and fusion, *n* (%)	25 (5.2)
Age at posterior fusion, year, mean ± SD	14.4 ± 2.3
Age at anterior fusion, year, mean ± SD	14.9 ± 2.1

Orthosis prescription data was extracted from 2016–2018. SD, standard deviation.

With regard to orthopedic management, there were 22 (4.6%) and 9 (1.9%) patients from 2016 to 2018 with ankle–foot orthosis and spine orthosis, respectively, as could be verified from the insurance benefits data. Moreover, 77 (16.1%) patients had posterior spinal instrumentation and fusion at 14.4 ± 2.3 years, and 25 (5.2%) had anterior spinal instrumentation and fusion at 14.9 ± 2.1 years (Table 2B).

## Discussion

DMD is the most common subtype of MD with severe clinical symptoms. Patients with DMD show progressive muscle and respiratory weakness, loss of ambulation and have a median life expectancy with ventilatory support ranging from 21.0 to 39.6 years^5^. In this study, the mean mortality age shows an increasing trend every year, and this trend is thought to reflect the situation when the implementation of NIV for DMD patients was started in South Korea. In addition, this study found that the survival rate of adult DMD patients over the age of 20 has shown an increasing trend and the age of mortality has continuously increased. Most clinicians are aware of the benefits of glucocorticoid therapy to improve life expectancy and preserve respiratory function^13^. To the best of our knowledge, this is the first database study to investigate the current status of corticosteroid and related bone health management directly related to disease progression in patients with DMD in South Korea.

### Glucocorticoid therapy

Long-term steroid use in patients with DMD has prolonged life expectancy and changed the overall natural history^14^, although their prescription has still not been standardized^15^. According to the results of the present study, steroid prescription is mostly not initiated at the time of DMD physical decline; this is inconsistent with the findings of recent studies emphasizing the benefits of early initiation of steroid therapy before the onset of physical decline^16^. In our study, deflazacort tended to be increasingly prescribed to younger patients compared with prednisolone; however, the age of initiation did not decrease over the years. Although the benefits of glucocorticoid therapy are well-established, considering the benefit-to-risk ratio of the drugs, uncertainty remains regarding the appropriate regimen and the use of steroid therapy after the loss of ambulation. According to the survey of TREAT-NMD network, 29 different corticosteroid regimens were used worldwide. Nevertheless, the long-term outcomes of the many different regimens remain unclear^17^. Although it has been reported that glucocorticoid therapy is effective in terms of maintaining upper-limb function and cardiorespiratory function^17^, the long-term use of steroids can cause various side effects, such as bone health problems, obesity, and behavioral changes^18^, and consensus has not been reached regarding maintaining the prescription of steroids during the non-ambulant period. On examining the drug prescription regimen data collected in this study, it was confirmed that the percentage of non-ambulant patients taking steroids increased by the year. According to the DMD care considerations^10^, it is recommended to continue steroids in non-ambulatory patients, and steroid-naïve older patients might benefit from the introduction of steroids^9^. This aligns well with the results of our study.

Previous studies have compared the superiority between prednisolone and deflazacort; however, it is difficult to draw a definite conclusion from these results. Deflazacort is a glucocorticoid derived from prednisolone and 6 mg of deflazacort had approximately the same anti-inflammatory potency as 5 mg prednisolone. The benefit-to-risk ratio of deflazacort compared with prednisone is being studied further in an ongoing double-blind trial^19^.

Based on the data of this study, the percentage of deflazacort prescriptions was similar to that of prednisolone in recent years. Although the deflazacort prescription dose in the ambulant group increased by age and year, the dose prescription for both deflazacort and prednisolone did not reach the recommendation of the current DMD care consideration (prednisolone: 0.75 mg/kg per day; deflazacort: 0.9 mg/kg per day). Analysis of more than 3000 patients in the Duchenne Registry revealed that the recommended dose was not administered^20^. Thus, for appropriate steroid prescription, a consensus should be established among clinicians globally, including those in South Korea. In our study, the mean age of patients prescribed with deflazacort as initial corticosteroid was lower than that of patients prescribed with prednisolone. As one previous study reported that initial corticosteroid was prescribed to patients with a mean age of 5 years in the DMD registry, discussions on the timing and dose of prescription considering the benefits and disadvantages of steroids are continuously needed.

In addition, considering that the recent clinical trials for new medicine for DMD treatment are excluding the patients not receiving previous corticosteroid therapy as the recommended regimen, discussion among clinicians about appropriate prescription of the corticosteroid should be discussed further.

### Bone health management

With regard to glucocorticoid-treated DMD cases, there is a high incidence of glucocorticoid-induced osteoporosis, and the resulting bone fragility may lead to secondary vertebral and long-bone fractures^21^. In addition, after non-ambulatory stage, immobilization, such as full-time wheelchair use, effects on bone calcium homeostasis and the risk for fracture by casing additional demineralization of bone.

In the current DMD care considerations^10^, serial spine radiography is recommended over DXA scan to determine asymptomatic bone fragility. In a recent expert study, DXA was the most common method, followed by spine X-rays, and biochemical marker assessment. In addition, more than 61% of clinicians answered that they implemented a routine bone health assessment^11^. In the present study, among the patients assessed for bone health monitoring, less than 30% underwent spine radiography, DXA, scan, and Vitamin D level examination. DXA was confirmed to be the most commonly used method among all tests. All tests were conducted with longer time intervals than the recommendation. Thus, we need to consider whether additional regular bone health screening is required according to the recommendation. Approximately 40% and 10% of patients were prescribed vitamin D and bisphosphonate; however, considering the additional needs for DXA scan based on our results, the percentage of patients requiring the prescription may also increase.

### Orthopedic management

Orthopedic management of DMD is necessary to minimize joint contractures and prolong ambulatory function as much as possible. A custom-molded night-time ankle–foot orthosis prescription can be used from the ambulatory stage to delay the progression of the equinovarus contracture of the ankle and extend the ambulatory stage; even if the patient becomes wheelchair-bound, it is possible to assume a proper sitting posture by maintaining the ankle joint. In this study, approximately 5% of the patients were given prescriptions, and as the study data only included 3 years of available benefits payment table records, the actual prescription rate is presumed to be higher. With the onset of the non-ambulatory stage, scoliosis progresses rapidly, causing discomfort in the sitting posture; this can also lead to compromised respiratory function^22^. Therefore, monitoring of radiography assessment annually or every 6 months after the confirmatory diagnosis of scoliosis is recommended^10^. According to the result of our study, the assessment frequency in the applicable age group was judged to be longer than expected.

There is low-level evidence that spinal orthoses can delay the progression of scoliosis^10^. However, there were cases in which spinal orthosis was prescribed, assuming that the initial mobile curve could be corrected and maintained with the aid of an orthosis^23^. Although we confirmed that it was prescribed to some patients in our study, the result was analyzed during the 3 years for which data were available so the actual prescription rate can be presumed to be higher.

Surgery is recommended for functional improvement, sitting balance, and improvement of pain and the quality of life at a young age when a spinal curve of ≥ 20° has been measured^24,25^. Even with the consideration that corticosteroid therapy slows mild spinal curvature and reduces the necessity of spine surgery^26^, the results of our study confirmed that a low percentage of our patients underwent spinal surgery^27^, and the age of undergoing surgery was found to be higher than in the early teens, which is the age at which patients become wheelchair-bound. Otherwise, from the survey of the TREAT-NMD network, involving 5345 patients from the DMD global database, we recognize that approximately 10% of patients underwent scoliosis surgery. Since only half of these patients were under the age of 20, the age eligibility for surgical treatment according to the recent recommendation is not unique in South Korea, therefore requiring consensus among clinicians worldwide^17^. As posterior spinal instrumentation and fusion are recommended in non-ambulatory individuals with little additional longitudinal spine growth is anticipated^28^, the percentage of the posterior approach was three-time higher than the anterior approach in our data.

This study describes the current status of corticosteroid use and bone health management related to DMD, and it is the first study in a South Korean population in this regard.

There are some limitations to our study. First, we could not extract a complete list of patients with DMD from those with MD solely based on the diagnosis codes owing to the limitations of big data. In this study, we identified patients with MD who were prescribed corticosteroids and had the typical clinical course of DMD for analysis. Therefore, an epidemiological analysis could not be performed using the data collected in this study. However, DMD patients are the only subgroup of MD patients who are prescribed steroids, and considering the recent guidelines for early prescriptions and maintenance doses, it was presumed that relatively few patients would have been excluded. In addition, we only extracted and analyzed cases designated as DMD that was prescribed steroid,’ which is considered to be more reliable. Furthermore, as many patients with DMD are diagnosed based on non-covered genetic testing instead of a muscle biopsy or EMG as a result of advances in diagnostic techniques, we could not perform an epidemiological analysis encompassing incidence and time of diagnosis. Thus, it is important to establish a nationwide DMD registry in Korea to examine the clinical course and management status of DMD and reach a consensus. Second, with regard to orthosis prescription, information could only be obtained for 3 years, when payment based on the benefits payment table was possible. A further study that can analyze long-term data will be useful to understand the current status of DMD management in greater detail. Lastly, Due to the nature of this study based on large-scale data from the National Health Insurance Service (NHIS), we could identify the prescription details of the examinations or treatment. However, it was impossible to confirm the results of these examinations because this could expose personally identifiable information.

## Conclusion

In this our study, the current corticosteroid use and bone health management status of DMD in South Korea has been presented. The dose prescription for corticosteroid and bone health monitoring did not reach to current recommendation. In order to adequately monitor the changing clinical practices in South Korea, it is expected that the findings of this study will contribute to raising awareness on the necessity of establishing a domestic registry in the country for patients with DMD and of developing consensus among clinicians in the long term.

## Data Availability

The datasets generated and/or analysed during the current study are not publicly available as current study are from the Korean National Health Insurance which is not publicly available due to participant privacy concern but are available from the corresponding author on reasonable request.

## Abbreviations

DMD: Duchenne muscular dystrophy
DXA: Dual-energy X-ray absorptiometry
